# Development of a New Model System to Study Long-Distance Interactions Supported by Architectural Proteins

**DOI:** 10.3390/ijms25094617

**Published:** 2024-04-23

**Authors:** Larisa Melnikova, Varvara Molodina, Pavel Georgiev, Anton Golovnin

**Affiliations:** 1 Department of Drosophila Molecular Genetics, Institute of Gene Biology, Russian Academy of Sciences, 34/5 Vavilov Street, Moscow 119334, Russia; molodina_varvara@mail.ru; 2 Department of the Control of Genetic Processes, Institute of Gene Biology, Russian Academy of Sciences, 34/5 Vavilov Street, Moscow 119334, Russia; georgiev_p@mail.ru

**Keywords:** long-distance interactions, insulator, architectural C2H2 proteins, Su(Hw), CP190, Mod(mdg4), enhancer–promoter communication

## Abstract

Chromatin architecture is critical for the temporal and tissue-specific activation of genes that determine eukaryotic development. The functional interaction between enhancers and promoters is controlled by insulators and tethering elements that support specific long-distance interactions. However, the mechanisms of the formation and maintenance of long-range interactions between genome regulatory elements remain poorly understood, primarily due to the lack of convenient model systems. *Drosophila* became the first model organism in which architectural proteins that determine the activity of insulators were described. In *Drosophila*, one of the best-studied DNA-binding architectural proteins, Su(Hw), forms a complex with Mod(mdg4)-67.2 and CP190 proteins. Using a combination of CRISPR/Cas9 genome editing and *attP*-dependent integration technologies, we created a model system in which the promoters and enhancers of two reporter genes are separated by 28 kb. In this case, enhancers effectively stimulate reporter gene promoters in *cis* and *trans* only in the presence of artificial Su(Hw) binding sites (SBS), in both constructs. The expression of the mutant Su(Hw) protein, which cannot interact with CP190, and the mutation inactivating Mod(mdg4)-67.2, lead to the complete loss or significant weakening of enhancer–promoter interactions, respectively. The results indicate that the new model system effectively identifies the role of individual subunits of architectural protein complexes in forming and maintaining specific long-distance interactions in the *D. melanogaster* model.

## 1. Introduction

In higher eukaryotes, many genes, especially those regulating development, have complex regulatory regions containing dozens of enhancers, which in some cases are located at a distance of hundreds of kb from the target promoters [1,2,3,4]. Chromosomes of higher eukaryotes are organized into large topologically associating domains (TADs), where long-distance interactions between regulatory elements occur more frequently than between neighboring TADs [5,6]. According to current concepts, insulators and a recently discovered new class of regulatory elements, called tethering elements, play essential roles in forming functional long-distance interactions between enhancers and promoters [7,8].

The first evidence that interactions between insulators can support long-distance interactions came from a study in which the pairs of *gypsy* or *Mcp* insulators supported functional interactions between two Polycomb response elements (PREs) located in transposons inserted over distances that sometimes exceeded one megabase [9,10,11]. It has also been shown that super-long-distance interactions can be supported by the Fab-7 boundary, which separates the domains of the *Abdominal-B* regulatory region in the Bithorax complex [12]. The most well-studied interaction is between the Neighbor of Homie (Nhomie) insulator and the Homing insulator at *eve* (Homie), which are located at the boundaries of 16 kb *even skipped* (*eve*) regulatory region [13,14,15]. Nhomie and Homie interact with each other and can also support a super-long-distance interaction between the transgene and the endogenous *eve* locus, which allows endogenous enhancers for the activation of the reporter gene promoter in the transgene [14,15,16].

The group of architectural proteins responsible for the functions of known insulators and involved in maintaining specific long-distance interactions has been well-studied in *Drosophila* [17]. The architectural protein Su(Hw), one of the first to be described, contains 12 C2H2 zinc fingers (C2H2 domains) and binds to 12 sites in the 5′ regulatory region of the *gypsy* retrotransposon [18,19,20,21,22]. C2H2 domains from 6 to 10 are involved in specific binding to the 15 bp DNA motif [23]. Other studied architectural proteins, especially CTCF and Pita, also contain clusters of C2H2 domains [24]. All described *Drosophila* architectural proteins, except Su(Hw), have N-terminal domains that can form homodimers or multimers and help maintain specific long-distance contacts in transgenic lines [17].

One feature of *Drosophila* architectural C2H2 proteins is their direct interaction with the CP190 protein [17,25,26]. CP190 predominantly associates with gene promoters and is required to support an open chromatin structure [27,28,29,30,31]. Several studies have suggested that CP190 is responsible for the association of NURF, dREAM, SAGA, and other complexes with promoters [32,33,34,35]. The CP190 protein binds to all boundaries in the Bithorax complex and helps organize independent transcriptional domains [36,37,38].

Two conserved regions at the N-terminus of the Su(Hw) protein interact with the Broad-complex, Tramtrack, and Bric-à-brac (BTB) domains of the CP190 protein [25,39,40]. At the C-terminus of Su(Hw), the mapped region interacts with one of more than 30 isoforms of the Mod(mdg4) protein [41,42,43,44]. All Mod(mdg4) isoforms at the N-terminus of the protein have a Tramtrack (TTK)-like BTB domain, which can form hexamers [45,46]. The Mod(mdg4)-67.2 isoform has a unique C-terminus that interacts exclusively with the C-terminal domain of the Su(Hw) protein [42,43,44]. The CP190 and Mod(mdg4)-67.2 proteins are simultaneously required for the activity of the Su(Hw)-dependent complex and its recruitment to chromatin [44]. Su(Hw)-dependent complexes may also include other proteins, such as HIPP1 [47,48,49,50], CP60 [51,52], and RNA-binding proteins [53,54,55]. Several studies have suggested the participation of the CP190 and Mod(mdg4)-67.2 proteins in maintaining long-distance interactions [56,57,58,59]. However, direct experimental evidence of the role of these proteins in organizing distance contact has not yet been obtained. Previously, the role of architectural proteins and their individual domains in managing specific interactions between regulatory elements and in insulation was studied using a transgenic system consisting of two tandemly located reporter genes, *yellow* and *white* [60]. However, this model system did not allow the study of functional interactions between regulatory elements over long distances.

This study established a new model system in which functional interactions between enhancers and promoters at a distance can be determined by phenotypic analysis of the pigmentation intensity of the body cuticle and eyes. As an example, it was shown that CP190 and Mod(mdg4)-67.2 proteins, which are recruited to Su(Hw) binding sites, can support functional long-distance interactions between enhancers and promoters. In general, the model system makes it easy to assess the possibility of long-range interactions between protein complexes formed by identical or different architectural proteins associated with their distantly located sites.

## 2. Results

### 2.1. Generation of a Model System for Testing Long-Distance Interactions between Regulatory Elements

To create our model system, we chose a 2R chromosome extended region from 7735 to 7780 kb (approximately 45 kb), enclosed in one TAD (Figure S1). This region, along its entire length, is enriched with H3K36me3, with gradual growth of H3K79me3, H3K4me3, and H3K4me1 histone marks closer to the TAD border. Such structure reflects the transcriptionally active unrepressed TAD [61,62]. It does not contain endogenous binding sites for known architectural/insulator proteins. To integrate the model constructs, we used the *CG34230/Tweedlβ* locus (2R:11862296) (hereafter called site A) and the large intron of the *ERp60* gene (2R:11890256) (hereafter called site B), located at a distance of 28 kb from each other (Figure S1).

**Figure 1:**
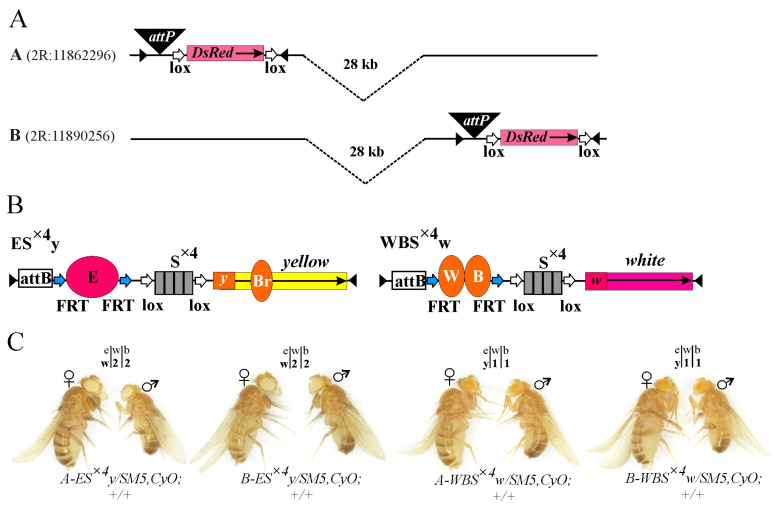
Integrated constructs and control phenotypes. (**A**) Schematic representation of *attP* sites integrated into selected loci on homologous chromosomes and separated by 28 kb. The *loxP* sites are designated by horizontal arrows, and the pink box indicates the *DsRed* fluorescent marker. (**B**) Schematic representation of integrated constructs ES^×4^y and WBS^×4^w. Enhancers of eye (E), body (B), wings (W), and bristles (Br) are represented by ovals. The *FRT* (Flp recognition target) and *loxP* sites are indicated by blue and white horizontal arrows, respectively. Promoters of *yellow* (*y*) and *white* (*w*) genes are shown as squares. The direction of gene transcription is indicated by black horizontal arrows. The four grey boxes represent the four Su(Hw) binding site multiplicities (S^×4^). White rectangles represent the *attB* integration sites. The *yellow* and *white* genes are indicated. (**C**) Photo showing the representative phenotypes of flies, with the corresponding constructs (marked below the photo) inserted in either the A or B genome locus. Numbers above the photo indicate the scores of *white* and *yellow* gene expression in the eyes (e), wings blades (w), and body cuticle (b). Scales of eye and abdominal pigmentation are presented in Figure S2A.

Using CRISPR/Cas9 genome editing, we integrated *attP* sites into two independent *y^1^w^1118^* lines, with null alleles for *yellow* and *white* genes at position A or B. The expression of the *DsRed* gene was used as a marker of successful integration. The *DsRed* gene was then excised using Cre–Lox recombination (Figure 1A) [63]. All obtained transgenic lines were viable and fertile.

Two constructs were created to test the ability of the architectural Su(Hw) protein to support long-distance interactions. Each construct includes an *attB* site [64,65] and an artificial DNA sequence consisting of four Su(Hw) protein binding motifs (S^×4^) [66], flanked by *loxP* sites [63]. In one construct, the *yellow* gene was used as a reporter, which is responsible for the body and wing cuticle pigmentation [67]. In the other, the *white* gene provides eye pigmentation (Figure 1B) [68]. Inactivation of the *yellow* and *white* genes does not affect survival, and their expression levels can be determined visually with high accuracy. In the body cuticle and wing blades, *yellow* transcription is controlled by enhancers located at a distance of 1100 bp from the promoter [67]. They determine the dark color of the body and wings and the black color of the last two abdominal segments (A5 and A6) in males. In the absence of *yellow* gene enhancers or when they are blocked by the *gypsy* insulator in the *y^2^* allele [69], the body and wings are yellow, and the two abdominal segments of males become light brown. The degree of pigmentation of the body and wings directly correlates with the activation efficiency of the *yellow* gene by the enhancers and is assessed on a scale from 1 to 5 (Figure S2A) [60]. Transcription of the *white* gene in the eye imaginal disc is controlled by an eye-specific enhancer located 1150 bp upstream of the promoter [68]. Without its eye-specific enhancer, basal *white* gene expression in transgenes typically ranges from yellow to orange. In the presence of the enhancer, increased expression of the *white* gene correlates with increased eye pigmentation to dark brown or red (Figure S2A).

We used the standard *mini-white* (hereafter referred to as *white*) gene, which has the first intron deleted. The body (B) and wing (W) enhancers were placed in the WBS^×4^w construct, marked with the *white* reporter (w), and the eye-specific enhancer (E) was placed in the ES^×4^y construct, marked with the *yellow* (y) reporter. In both constructs, the enhancers were flanked by *FRT* sites (Figure 1B) [70].

The constructs were independently integrated into the genome of the *y^1^w^1118^*-A and *y^1^w^1118^*-B lines (Figure S2B) using *φC31* recombinase [64,65]. At both integration sites (sites A and B for each transgene), the body and wing cuticle of *ES^×^*^4^*y/SM5*,*CyO;+/+* flies were weakly pigmented, while the eyes of the *WBS^×^*^4^*w/SM5*,*CyO;+/+* flies were yellow, corresponding to the basal level of reporter transcription. Thus, in the genome regions selected for integration, the surrounding chromatin did not affect the transgene expressions (Figure 1C).

### 2.2. Artificial Su(Hw) Protein Binding Sites Can Support Long-Distance Interactions between Enhancers and Promoters of Reporter Genes in the Model System

**Figure 2:**
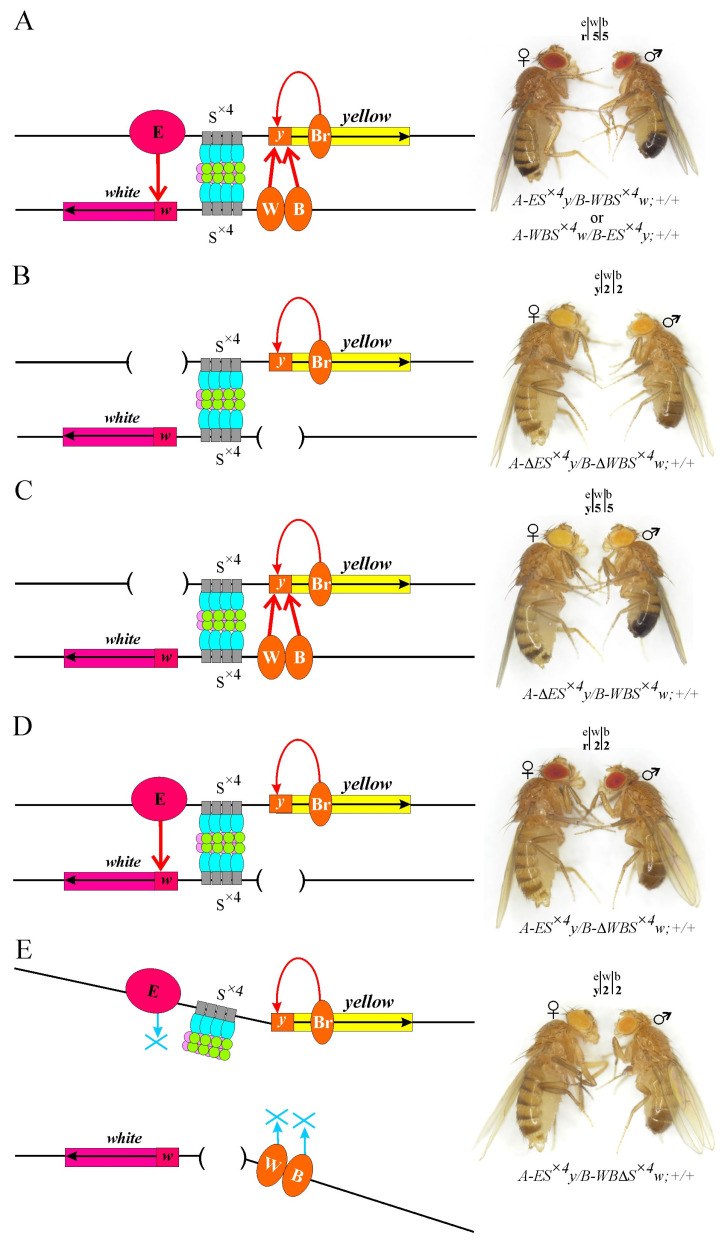
Trans interactions between enhancers and promoters of reporter genes in the model system. (**A**–**E**) Schematic representations of interacting transgenes. Thin red arrows indicate transcriptional activation by specific enhancers, and the blue arrow with a cross indicate the loss of the enhancer–promoter interaction. The Su(Hw) (blue ovals), CP190 (green circles), and Mod(mdg4)-67.2 (pink circles) proteins are designated. Brackets represent the deletion of the corresponding elements in the constructs. The photos show the representative phenotypes of the flies with a combination of corresponding constructs (marked below each photograph). Other designations are as in Figure 1.

To demonstrate the ability of enhancers to activate reporter promoters located *trans* at a distance of 28 kb, we obtained trans heterozygotes *A-ES^×^*^4^*y/B-WBS^×^*^4^*w;+/+* and *B-ES^×^*^4^*y/A-WBS^×^*^4^*w;+/+*. In both lines, the expression levels of the *yellow* and *white* genes were similar to that of the wild type (wt) (Figure S2B). The body cuticle and wings were dark, and the eyes were bright red (Figure 2A), which suggests a functional trans interaction between enhancers and reporter gene promoters located at a distance of 28 kb. Moreover, the activity of enhancers did not depend on the insertion site of the corresponding constructs (Figure 2A).

To experimentally confirm the role of enhancers in reporter activation, we deleted the eye-specific enhancer (ΔE) from the *A-ES^×^*^4^*y/SM5*,*CyO;+/+* line and the body and wing enhancers (ΔWB) from the *B-WBS^×^*^4^*w/SM5*,*CyO;+/+* line using Flp–FRT recombination. The phenotype of the resulting *A-*Δ*ES^×^*^4^*y/B-*Δ*WBS^×^*^4^*w;+/+* trans heterozygotes corresponded to the basal level of expression of the *yellow* (yellow body and wings, light brown abdominal segments) and *white* (yellow eyes) genes (Figure 2B).

In the trans heterozygotes *A-*Δ*ES^×^*^4^*y/B-WBS^×^*^4^*w;+/+* with deletion of only the eye-specific enhancer, the expression of the *white* gene remained at the basal level (yellow eyes). At the same time, high-level *yellow* gene expression was observed (dark body and wings, black abdominal segments) (Figure 2C). In contrast, in trans heterozygotes *A-ES^×^*^4^*y/B-*Δ*WBS^×^*^4^*w;+/+*, only the *white* gene expression (red eyes) was activated (Figure 2D). Therefore, in the model system, enhancers independently activate specific promoters only, and S^×4^ elements do not affect reporter transcription.

To test the role of SBS in organizing long-distance enhancer–promoter interactions, the S^×4^ element was deleted (ΔS^×4^) from the *B-WBS^×^*^4^*w;+/+* line using Cre–Lox recombination. The derived *B-WB*Δ*S^×^*^4^*w/SM5*,*CyO;+/+* line was crossed with the *A-ES^×^*^4^*y/SM5*,*CyO;+/+* line. The absence of even one of the S^×4^ sites in trans heterozygotes *A-ES^×^*^4^*y/B-WB*Δ*S^×^*^4^*w;+/+* results in a reduction in the *yellow* and *white* gene transcription to the basal level (Figure 2E). Thus, the deletion of S^×4^ leads to a loss of the functional interaction between remote enhancers and reporter gene promoters, demonstrating the crucial role of SBS in organizing long-distance interactions.

### 2.3. Testing the Combined Roles of Mod(mdg4)-67.2 and CP190 in Long-Distance Interactions

The Su(Hw) protein was previously shown to bind to S^×4^ sites and recruit CP190 and Mod(mdg4)-67.2 proteins to chromatin [66]. To reveal the role of CP190 and Mod(mdg4) in maintaining long-distance enhancer–promoter interactions in our model system, we alternately inactivated the recruitment of each protein to the S^×4^ site. Using a two-step genome engineering platform that combines CRISPR with the *φC31/attP* recombination system [71], we created a null allele Δ*su(Hw)*, where the *su(Hw)* gene was replaced by the *DsRed* reporter gene and the *attP* site (Figure S3A). We obtained one line with the null Δ*su(Hw)* mutation, which was confirmed by the polymerase chain reaction (PCR) (Figure S3B). As in previously described *su(Hw)* null mutations [72], Δ*su(Hw)* mutants survived as homozygotes, but the females were sterile. The Δ*su(Hw)* mutation suppressed insulation in the *y^2^* [69] and *ct^6^* [43] alleles (Figure S3C). Western blot analysis with the control *y^2^w^1118^ct^6^* (wt) line and the line carrying the Δ*su(Hw)* allele showed that anti-Su(Hw) antibodies recognize a 130 kDa Su(Hw) protein in the wt, but not in the *y^2^w^1118^ct^6^*;Δ*su(Hw)/*Δ*su(Hw)* line (Figure S3D). Null mutations in the *Cp190* gene are lethal at the pupal stage [73], making phenotypic analysis at the adult stage impossible. Therefore, using the *φC31/attP* recombination system, we integrated two independent transgenes into the Δ*su(Hw)* allele, one of which expressed the Su(Hw)Δ114 protein, with a deletion of the N-terminal region that interacts with the CP190 protein (88–202 aa) [40]. In contrast, another one expressed the full-length Su(Hw)FL protein. Both proteins were tagged with the FLAG tag and expressed under the control of the *ubiquitin-63E* promoter. Western blot analysis with anti-FLAG antibodies was used to confirm the equal expression of the Su(Hw) variants (Figure S3D). In the *y^2^w^1118^ct^6^;su(Hw)^FL^/su(Hw)^FL^* line, insulation in the *y^2^* and *ct^6^* alleles was completely restored, whereas in the *y^2^w^1118^ct^6^;su(Hw)*^Δ*114*^*/su(Hw)*^Δ*114*^ line, it was partially restored (Figure S3C). The phenotype in these lines was the same as in previously described lines, where the corresponding transgenes were inserted at the 38D site (Figure S3C) [40].

**Figure 3:**
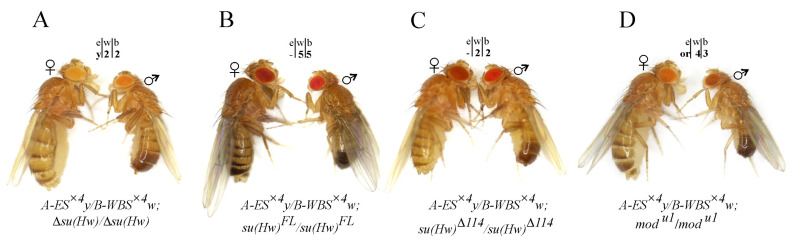
Fly phenotypes (**A**–**D**) from the *A-ES^×^*^4^*y/B-WBS^×^*^4^*w* line with different mutant backgrounds. All designations are as in Figure 1. Sign “-” indicates that eye pigmentation was not scored.

Using genetic crosses, trans heterozygotes *A-ES^×^*^4^*y/B-WBS^×^*^4^*w* were obtained in combination with Δ*su(Hw)*, Su(Hw)FL, or Su(Hw)Δ114 expressing transgenes. Because the expressing transgenes were marked with the *white* gene, the enhancer–promoter interactions were analyzed for *yellow* expression only. With the knockout of the Su(Hw) protein (Δ*su(Hw)*) (Figure 3A), when the insulator complexes were not formed at the S^×4^ sites, the same as with the deletion of the S^×4^ element (Figure 2E), *yellow* expression remained at the basal level. Consequently, in the absence of Su(Hw) insulators, the remote enhancers were unable to interact with specific promoters and activate the transcription of the reporter genes. The introduction of the *su(Hw)^FL^* transgene in the model system increased *yellow* expression to the wild-type level (Figure 3B). In this case, the expressed Su(Hw)FL protein recruited Mod(mdg4)-67.2 and CP190 to the S^×4^ sites and formed a Su(Hw)-dependent complex. Thus, the interaction of the insulator complexes ensured the functional interaction between enhancers and promoters in this model system.

In the *A-ES^×^*^4^*y/B-WBS^×^*^4^*w;su(Hw)*^Δ*114*^*/su(Hw)*^Δ*114*^ line, where the CP190 protein did not bind to the Su(Hw)-dependent complex, flies had a yellow body and wings (Figure 3C), demonstrating a complete absence of enhancer–promoter interactions. Therefore, the CP190 protein is critical for long-distance interactions between the Su(Hw)-dependent complexes formed at the S^×4^ sites. To study the functional role of the Mod(mdg4)-67.2 protein, we combined the trans heterozygote *A-ES^×^*^4^*y/B-WBS^×^*^4^*w* with the homozygous mutation *mod(mdg4)^u1^*. The *mod(mdg4)^u1^* mutation does not affect viability and completely inactivates the Mod(mdg4)-67.2 isoform only, which specifically interacts with Su(Hw) [44]. In the *A-ES^×^*^4^*y/B-WBS^×^*^4^*w;mod(mdg4)^u1^/mod(mdg4)^u1^* line, the flies had orange eyes and dark wings. However, the pigmentation of the male abdominal segments remained similar to the basal level (Figure 3D). Thus, upon the inactivation of the Mod(mdg4)-67.2 protein, distant enhancers partially retain the ability to activate the expression of both reporters. One can assume that in organizing long-distance interactions between the S^×4^ sites, the CP190 protein plays a more important role than Mod(mdg4)-67.2. Alternatively, the absence of the CP190 protein strongly destabilizes the binding of the Su(Hw)-dependent complex to chromatin. The data from the phenotypic analysis of the reporter gene expression in the lines of the model system are summarized in Figure S4.

### 2.4. The Level of Insulator Proteins Binding to S×4 Confirms Their Role in Long-Distance Interactions

**Figure 4:**
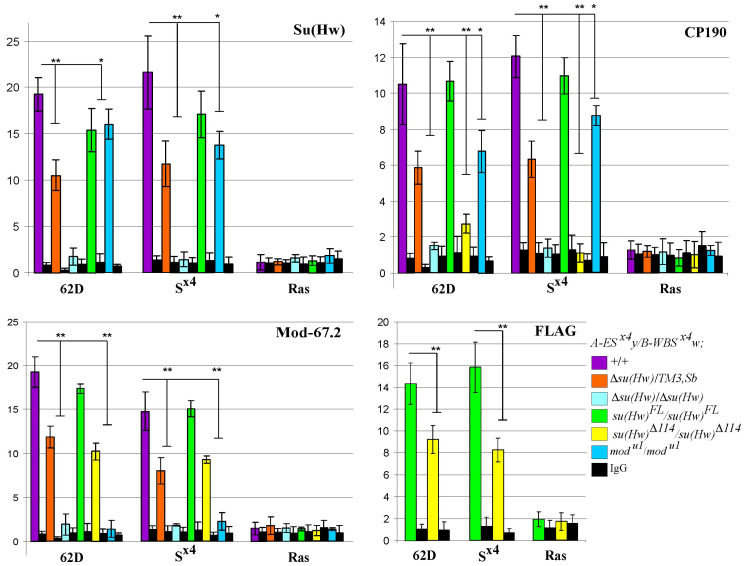
ChIP–qPCR analysis of protein binding to the S^×4^ site in the *A-ES^×^*^4^*y/B-WBS^×^*^4^*w* line with different mutant backgrounds. The Su(Hw) binding in the *su(Hw)*^Δ*114*^*/su(Hw)*^Δ*114*^ mutant background was detected by anti-FLAG antibodies because antibodies related to the N-terminal domain of Su(Hw) were not detect truncated Su(Hw). Tested mutant backgrounds are indicated by colored boxes, with designations as in Figure 3. IgG, immunoprecipitation with nonspecific IgG. The 62D and *ras64B* coding regions (Ras) were used as positive and negative controls, respectively. The percentage recovery of immunoprecipitated DNA (y-axis) was calculated relative to the amount of input DNA. Error bars indicate standard deviations of quadruplicate polymerase chain reaction (qPCR) measurements from two independent biological samples of chromatin. Asterisks indicate significance levels: * *p* < 0.05 and ** *p* < 0.01 (Student’s *t*-test).

The CP190 and Mod(mdg4)-67.2 proteins are required for the efficient recruitment of the Su(Hw)-dependent complex to chromatin [44]. Therefore, long-distance interactions between insulators may be disrupted due to the absence of CP190 or Mod(mdg4)-67.2 proteins in the complex or by the decreased binding of the entire complex to S^×4^ sites. To select between these two possibilities, we tested the binding of components of the Su(Hw)-dependent complex to S^×4^ elements in the model system lines using ChIP–qPCR analysis. The previously characterized SBS 62D [74] and the SBS-free *ras64B* gene sequence were used as internal controls. As expected, in the *A-ES^×^*^4^*y/B-WBS^×^*^4^*w*;*su(Hw)^FL^/su(Hw)^FL^* line, the level of insulator protein binding was comparable to the wild type, whereas in the Su(Hw) knockout (Δ*su(Hw)*/Δ*su(Hw*)), none of the proteins were recruited to the S^×4^ site (Figure 4).

In the *A-ES^×^*^4^*y/B-WBS^×^*^4^*w;mod(mdg4)^u1^/mod(mdg4)^u1^* line, the level of Su(Hw) and CP190 protein binding decreased by 25–30% (Figure 4). The Su(Hw) binding in the *A-ES^×^*^4^*y/B-WBS^×^*^4^*w;su(Hw)*^Δ*114*^*/su(Hw)*^Δ*114*^ line was tested using antibodies related to the FLAG epitope. This was because, in the Su(Hw)Δ114 derivative, the 1–150 aa region specifically recognized by antibodies related to the N-terminal domain of Su(Hw) was partially deleted. The binding of the Su(Hw)Δ114 protein to the S^×4^ site was reduced compared with the binding of the full-length Su(Hw)FL protein. The Mod(mdg4)-67.2 protein binding decreased proportionally to Su(Hw) binding (Figure 4). In addition to the direct involvement of the Mod(mdg4)-67.2 and CP190 proteins in maintaining functional enhancer–promoter interactions, it is also possible that the level of Su(Hw) protein enrichment at S^×4^ sites may affect the maintenance of such interactions.

To test how a slight reduction in the Su(Hw) protein binding to the S^×4^ sites could affect functional interactions between the enhancers and promoters in the model system, we used the *A-ES^×^*^4^*y/B-WBS^×^*^4^*w;*Δ*su(Hw)/TM3*,*Sb* line in which Su(Hw) protein expression was decreased by 50% compared to the wild type (Figure S3D). We observed a clear decrease in the binding of Su(Hw), CP190, and Mod(mdg4)-67.2 proteins to the S^×4^ site compared to the wild type (Figure 4). However, in contrast to the *su(Hw)*^Δ*114*^ and *mod(mdg4)^u1^* mutations, reducing the Su(Hw) expression in the Δ*su(Hw)/TM3*,*Sb* heterozygotes did not affect the long-distance functional enhancer–promoter interactions in the model system.

Phenotypically, *A-ES^×^*^4^*y/B-WBS^×^*^4^*w;*Δ*su(Hw)/TM3*,*Sb* flies did not differ from *A-ES^×^*^4^*y/B-WBS^×^*^4^*w;*+/+ and *A-ES^×^*^4^*y/B-WBS^×^*^4^*w*;*su(Hw)^FL^/su(Hw)^FL^* flies (Figures S4 and S5). Consequently, the disruption of functional interactions in the lines of the model system in regard to the *mod(mdg4)^u1^*/*mod(mdg4)^u1^* and *su(Hw)*^Δ*114*^*/su(Hw)*^Δ*114*^ mutant backgrounds is caused by the absence of the Mod(mdg4)-67.2 or CP190 proteins, respectively, but not by the overall reduction in the Su(Hw)-dependent complex binding the S^×4^ site. Thus, both CP190 and Mod(mdg4)-67.2 proteins are required for effective long-distance interactions between Su(Hw) insulators.

### 2.5. Testing Long-Distance Cis Interactions

The trans interaction between enhancers and promoters on homologous chromosomes is unstable and depends on insulators that enhance pairing between homologous chromosomes [10,16,75,76,77,78]. Therefore, we determined whether S^×4^ sites are required for the functional interaction between enhancers and promoters of reporter genes located on the same chromosome.

**Figure 5:**
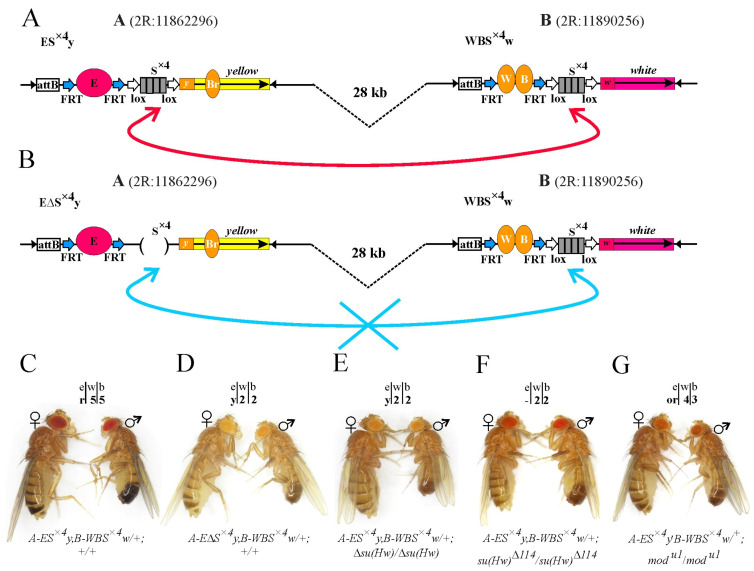
Model system for testing long-distance *cis* interactions, (**A**,**B**). (**C**–**G**) Fly phenotypes from the *A-ES×4y*,*B-WBS×4w/+* and *A-E*Δ*S×4y*,*B-WBS×4w/+* lines with different mutant backgrounds. All designations are as in Figure 1, Figure 2 and Figure 3.

We integrated transgenes expressing *yellow* and *white* genes into sites A and B on the same chromosome (Figure 5A,B). In the *A-ES^×^*^4^*y*,*B-WBS^×^*^4^*w/+;+/+* heterozygous line, where both transgenes contained an S^×4^ site (Figure 5A), the eyes, bodies, wings, and abdomen pigmentation were the same as in the trans heterozygotes *A-ES^×4^y/B-WBS^×4^w;+/+* (Figure 5C). Thus, the activation of *yellow* and *white* expression did not depend on whether the enhancers and promoters of the reporter genes were located on the same chromosome or homologous chromosomes. 

In the second heterozygous line *A-E*Δ*S^×^*^4^*y*,*B-WBS^×^*^4^*w/+;+/+*, where a transgene with a deleted S^×4^ element was integrated into site A (Figure 5B), activation of the reporter genes was at the basal level (Figure 5D). The introduction of the homozygous Δ*su(Hw)* mutation into the *A-ES^×^*^4^*y*,*B-WBS^×^*^4^*w/+* line also reduced the expression of reporters to the basal level (Figure 5E). Therefore, long-distance interactions between the enhancers and promoters of reporter genes in *cis* are supported by interactions between the S^×4^ sites.

Then, we analyzed the expression of *yellow* and *white* genes in the *A-ES^×^*^4^*y*,*B-WBS^×^*^4^*w/+* line in regard to the *mod(mdg4)^u1^/mod(mdg4)^u1^* and *su(Hw)*^Δ*114*^*/su(Hw)*^Δ*114*^ mutant backgrounds. The fly phenotypes were similar when the transgenes were localized on the homologous chromosomes. Without the CP190 protein in the Su(Hw)-dependent complex, the reporters were not activated (Figure 5F), and the absence of Mod(mdg4)-67.2 resulted in an intermediate phenotype (Figure 5G). Thus, in the created model system, both *trans* and *cis* interactions between the enhancers and promoters separated by a distance of 28 kb are ensured by Su(Hw)-dependent complexes formed at the S^×4^ sites.

## 3. Discussion

In this study, we demonstrated that two identical artificial DNA sequences containing four SBS (S^×4^) maintain the functional interactions between the enhancers and promoters of *yellow* and *white* reporter genes located at a distance of 28 kb on the same chromosome (in *cis*) or on homologous chromosomes (in *trans*). Such interactions provide transcription activation of both reporter genes by tissue-specific enhancers. These results are consistent with previous data demonstrating that two or three copies of S^×4^ regulate the functional interactions between the enhancers and promoters of *yellow* and *white* genes in a single transgene [60]. However, in previous studies, the regulatory elements were located at a distance of no more than 8 kb apart.

Our results also showed that interactions between Su(Hw) binding sites can support functional trans interactions between enhancers and promoters located on homologous chromosomes and separated by a distance of 28 kb. A well-described phenomenon in *Drosophila*, called transvection, concerns the ability of an enhancer or silencer located on one of the homologous chromosomes to activate or repress a promoter located on the second homologous chromosome [79,80,81,82]. Using transgenic systems, it has been shown that transvection occurs at most genomic sites [83,84,85]. A promoter located in close proximity to an enhancer can block its ability to trans stimulate another promoter located on a homologous chromosome and, thus, suppress transvection [82,86]. The efficiency of transvection between the enhancer and the promoter also generally decreases with increasing distance between their genomic positions and is highly dependent on the sites of integration of the constructs (position effect) [87]. Here, we found that S^×4^ sites can promote transvection by bringing the enhancers and promoters located in trans closer together in terms of space.

The model system developed in this study allows the transgene integration sites to be changed by varying the distance between the regulatory elements to study functional interactions at any distance. A helpful tool for introducing reporter constructs is the many (more than 100) available *attP* lines, which cover almost all *Drosophila* chromosomes. Using combinations of *attP* sites located at different distances in the same or different, active or repressed, TADs will allow testing of the protein interactions in different chromatin compartments.

Using the devised model system, we identified the role of the main components in the Su(Hw)-dependent complex, the Mod(mdg4)-67.2 and CP190 proteins, in organizing long-distance interactions. We showed that the deletion of the Su(Hw) domain responsible for CP190 binding leads to a complete loss of long-distance interactions between *yellow* enhancers and promoters. The critical role of the CP190 protein in maintaining long-distance interactions is consistent with data from recent genome-wide studies suggesting the participation of CP190 in organizing local contact between individual regulatory elements [88,89]. The CP190 protein contains the classical N-terminal BTB domain, which is conserved among higher eukaryotes and forms homodimers [25,46,90,91]. It is possible that CP190 homodimerization stabilizes the contact between Su(Hw)-dependent complexes or that CP190 recruits other yet unidentified proteins to SBS sites that mediate long-distance interactions.

Inactivation of the Mod(mdg4)-67.2 protein also partially reduces the efficiency of the functional interaction between enhancers and promoters of reporter genes. It has long been believed that the Mod(mdg4) protein plays a vital role in the organization of long-distance interactions and insulation [42,56]. The BTB domain of the Mod(mdg4)-67.2 protein forms hexamers and can interact with the BTB domains of other transcription factors from the TTK group [46], which can contribute to both maintaining distant contact and blocking interactions between enhancers and promoters. Because Mod(mdg4)-67.2 interacts with CP190 [44], these proteins may cooperatively participate in maintaining long-distance contact between SBS.

Previously, it was shown that identical insulators interact more effectively with each other than with heterological insulators [8]. That is the reason why there must be a mechanism that mediates the observed specificity of interactions between homologous protein complexes. It is believed that different combinations of proteins that bind in a second layer are responsible for such specificity. However, CP190 and Mod(mdg4) interact not only with the Su(Hw) protein, but also with many other DNA-binding proteins, including insulator proteins [40,44,92,93,94]. Therefore, these proteins are unlikely to be responsible for the specificity of the interactions between homologous protein complexes. The dimers of CP190 and the hexamers of Mod(mdg4) most likely stabilize already formed specific interactions. Previous data suggest that the specificity of long-distance interactions is determined by the homodimerization of DNA-binding proteins [8,17]. The specificity of the interactions between Su(Hw) insulators may be ensured by the Su(Hw) protein itself. However, the ability of Su(Hw) to form homodimers has not yet been studied experimentally.

Currently, many methods make it possible to detect the formation of chromatin loops between interacting regions at different levels of resolution in a cell population and individual cells [4,6,95]. Along with the widely used Hi-C technique, methods based on a combination of fluorescence in situ hybridization (FISH) staining with 3D modeling algorithms are rapidly developing, which make it possible to visualize the shape of single chromatin loops with unprecedented genomic resolution, study structural heterogeneity, and the dynamics of chromatin loop formation [96]. Compared to Hi-C, Micro-C identifies loops with higher resolution, including structure visualization. Based on this method, it was confirmed that in human cells, more than 50% of the loops are formed by insulators, while less than 10% of the loops are promoter–enhancer loops [97]. The single-cell imaging technology has highlighted how flexible and heterogeneous the 3D chromatin structure is, on the length scales relevant to transcriptional control [98]. However, lacking the measurements of transcription, it remains unclear which proximity metric is the most relevant for enhancer–promoter communication. Moreover, these approaches do not allow the identification of proteins and their domains involved in long-range contact. In this study, we created an effective model system that allows researchers to study the contribution of individual architectural proteins and their domains in maintaining functional interactions between regulatory elements located at large distances.

## 4. Materials and Methods

### 4.1. Plasmids and Cloning

To introduce an *attP* docking site in the *CG34230/Tweedlβ* locus (2R:11862296), we amplified a 1550 bp long upstream region with 5′ ttgaattcCAGCTTGCGAGTCCTGAGTT 3′and 5′ tttgcggccgcGGAAAGCAATAATTTATATTGC 3′ primers, and a 1684 bp long downstream region with 5′ ttagatctACTTGCGCAGTAACCTCATTTGT 3′ and 5′ ttctcgagGACATATTGTGAGCGAATTGCGA 3′ primers. Using EcoRI and NotI enzymes introduced in primers (underlined) for the amplification of the upstream region, we cloned this fragment in the p*DsRed–attP–loxP* plasmid using the same restriction sites. The resulting plasmid was digested by BglII and XhoI enzymes. The amplified downstream region was digested by BglII and XhoI enzymes, which were introduced in primers (underlined), followed by cloning in the prepared plasmid. The 5′ cttcGTTATTGGGTACCGATAATT 3′ and 5′ aaacAATTATCGGTACCCAATAAC 3′ primers were used to produce guide RNA.

To introduce an *attP* docking site in the *ERp60* locus (2R:11890256), we amplified a 1320 bp long upstream region with 5′ ttgaattcGTCTTGATTAGAGCCTCCCACT 3′ and 5′ tttgcggccgcACCCATAATCCCAGGAATGTG 3′ primers, and a 1830 bp long downstream region with 5′ ttggatccGCATAAGAAACTATAATAAAAGATG 3′ and 5′ ttctcgagGTTTGACACATGGTGTGACCGA 3′ primers. Using EcoRI and NotI enzymes introduced in primers (underlined) for the amplification of the upstream region, we cloned this fragment in the p*DsRed–attP*–*loxP* plasmid using the same restriction sites. The resulting plasmid was digested by BglII and XhoI enzymes. The amplified downstream region was digested by BamHI and XhoI enzymes, which were introduced in primers (underlined), followed by cloning in the prepared plasmid. The 5′ cttcGGATTATGGGTATAATGCAT 3′ and 5′ aaacATGCATTATACCCATAATCC 3′ primers were used to produce guide RNA.

To generate the null mutation of the *su(Hw)* gene (Δ*su(Hw)*), we amplified a 1500 bp long upstream region with 5′ ttgaattcGAATTCCAATTGTACGATTG 3′ and 5′ tccgcggGTGAGCGAGACAACACTAC 3′ primers, and a 1680 bp long downstream region with 5′ GCATGCGTACAATTACCGCAC 3′ and 5′ ttctcgagGAGCAGAAGCTGCGCAAGGT 3′ primers. Using EcoRI and SacII enzymes introduced in primers (underlined) for the amplification of the upstream region, we cloned this fragment in the p*DsRed–attP–loxP* plasmid using the same restriction sites. The resulting plasmid was digested by StuI and XhoI enzymes. The amplified downstream region was digested by the XhoI enzyme, which was introduced in the primer (underlined), followed by cloning in the prepared plasmid. The 5′ cttcGTGTTGTCTCGCTCACACCA 3′ and 5′ aaacTGGTGTGAGCGAGACAACAC 3′ primer pair was used to generate one guide RNA, while the 5′ cttcGTAATTGTACGCATGCATCA 3′ and 5′ aaacTGATGCATGCGTACAATTAC 3′ primer pair was used to produce another guide RNA.

To generate the WBS^×4^w construct, we amplified the enhancers that activate the *yellow* gene in the body cuticle and wing blades, located between −700 and −2873 bp relative to the transcription start site of the *yellow* gene, 2173 bp, by the 5′ TATGCAACTGACGATGGCTTAA 3′ and 5′ AATTGGAACTCGTGCTCG 3′ primers, followed by cloning in the p*BluescriptSK* (pSK) between two tandem *FRT* sites. Next, an *attB* site was cloned upstream to the *FRT* site, adjacent to the 5′ region of the enhancers. Multiplicated four times the third Su(Hw) binding site from the *gypsy* retrotransposon (S^×4^), flanked with two *loxP* sites, was cloned downstream of the *FRT* site, adjacent to the 3′ region of the enhancers. Finally, the *mini-white* gene (from −321 to +3190 bp), with a promoter region, was cloned next to the S^×4^ site.

To generate the ES^×4^y construct, we amplified the eye-specific enhancer from −1760 to −980, relative to the transcription start site of the *white* gene, 780 bp, by the 5′ TGTATGTAGGTGTGACTGTGC 3′ and 5′ GTCTCACTCAGCACTAGTAAC 3′ primers, followed by cloning in the pSK between two tandem *FRT* sites. Next, an *attB* site was cloned upstream of the *FRT* site, adjacent to the 5′ region of the enhancer. The S^×4^ site flanked with *loxP* sites was cloned downstream of the *FRT* site, adjacent to the 3′ region of the enhancer. Finally, the *yellow* coding region, with upstream regulatory sequences (from −700 to +4824 bp), was cloned next to the S^×4^ site.

To generate the EΔS^×4^y construct, we cut Su^×4^ from ES^×4^y with NotI and XbaI enzymes, then filled it in with the Klenow fragment and self-ligated. The Su(Hw)FL (UbqW-Su(Hw)1-945-FLAG) and Su(Hw)Δ114 (UbqW-Su(Hw)Δ114-FLAG) constructs were described earlier [40].

### 4.2. Drosophila Strains, Transformation, and Genetic Crosses

The flies were cultured on the standard yeast medium (water (distilled) 800 mL, agar 6 g, yeast 66 g, sucrose 66 g, semolina 30 g, raisins 30 g, propionic acid 5 mL) at 25 °C. Five females were mated with two males in vials and brooded every second day. The temperature and crowding were carefully controlled, as both factors affect pigmentation. We obtained the following stocks from the Bloomington Drosophila Stock Center (BDSC, Bloomington, IN, USA): y^1^M{w^+mC^=nos-Cas9.P}ZH-2Aw* (BDSC, stock No. 54591), y1w*;P{w+mW.hs=ET-FLPx2}291B/CyO (BDSC, stock No. 38885), *y^1^w^67c23^;sna^Sco^/CyO*,*P{w^+mC^=Crew}DH* (BDSC, stock No. 1092).

To obtain flies with the *attP* site in the *CG34230/Tweedlβ* locus (2R:11862296), or the *ERp60* locus (2R:11890256), *attP* docking constructs with the corresponding guide RNA were separately injected into the y^1^M{w^+mC^=nos-Cas9.P}ZH-2Aw* preblastodermal embryos. To obtain flies with deletion in the *su(Hw)* gene, the DNA of the reporter constructs was also injected into preblastodermal embryos with the y^1^ M{w^+mC^=nos-Cas9.P}ZH-2Aw* genotype. The resulting flies were crossed with *y^1^w^1118^* flies, and the transgenic progeny were identified by *DsRed* expression and verified by PCR analysis.

To obtain transgenic flies with the insertion of *su(Hw)^FL^* or *su(Hw)*^Δ*114*^ in the native genome region, the DNA of the reporter constructs was injected into preblastodermal embryos with the Δ*su(Hw)* genotype. The resulting flies were crossed with the *y^2^w^1118^ct^6^* flies, and the progeny carrying the transgene in the Δ*su(Hw)* region were identified by their eye color.

To obtain transgenic flies with the insertion of WBS^×4^w or ES^×4^y, the DNA of the reporter constructs was injected into preblastodermal embryos with the *attP* sites either in the *CG34230/Tweedlβ* (2R:11862296) or in the *ERp60* (2R:11890256) loci. The resulting flies were crossed with *y^1^w^1118^* flies, and the progeny carrying the transgene in the corresponding region were identified by their eye color or bristle pigmentation.

For the excision of the *DsRed* marker, the generation of lines with deletions of enhancers or the Su^×4^ element were obtained by crossing the flies bearing original constructs with the Flp (y^1^w^*^;P{w^+mW.hs^=ET-FLPx2}291B/CyO) or Cre (*y^1^w^67c23^;sna^Sco^/CyO*,*P{w^+mC^=Crew}DH*) recombinase-expressing lines. A high level of Flp recombinase was produced by exposing late embryos and second or third instar larvae to heat shock at 37 °C for 2 h. All excisions were confirmed by PCR analysis.

The construct Introduction into the homozygous *mod(mdg4)^u1^*, Δ*su(Hw)*, *su(Hw)^FL^*, or *su(Hw)*^Δ*114*^ backgrounds were performed using standard genetic crosses. The details of the crosses used for genetic analysis and for excision of the functional elements are available upon request.

### 4.3. Analysis of Yellow and White Phenotypes

Pigmentation of the wing blades and body cuticle in the abdominal segments was scored in 3 to 4 d old males by using a five-grade pigmentation scale [75], with pigmentation scores of 1 and 5 corresponding to the null phenotype and the wild-type, respectively. The *white* phenotype was estimated from the extent of eye pigmentation in adult flies (3 to 5 d old). Wild type, *white* expression determined the bright red eye color; in the absence of *white* expression, the eyes were white. Intermediate levels of *white* expression (in increasing order) were reflected in the eye color ranging from yellow through orange to brown. The pigmentation scores were independently determined by two investigators, who examined at least 50 flies from each transgenic line.

For quantification of pigmentation intensity, images were analyzed using the Measure tool of Fiji 1.54h. Measurements were performed using the images of males concerning the A5 abdominal segments or eyes, taken with the same settings. For each genotype, at least six representative images were processed. “Mode” parameters were used for histogram generation (Table S1).

### 4.4. Chromatin Preparation, ChIP Analysis, and Antibodies

Chromatin was prepared from flies at the adult stage, and the resulting chromatin preparation was used for the ChIP experiments, as described previously [99]. Immunoprecipitated DNA was quantified using qPCR with SYBR green (Bio-Rad, Hercules, CA, USA). The primers were positioned in the middle of the binding region, as identified in ModEncode by ChIP-seq. The primer sequences used in the qPCR for ChIP analyses are shown in Table S2. Analyses were performed on three biological replicates. Rabbit antibodies against CP190 (1:500), Su(Hw) (1:1000), Mod(mdg4)-67.2 (1:200), and mouse antibodies against FLAG (1:500) were used for the immunoprecipitations. Antibodies related to Su(Hw), CP190, and Mod(mdg4)-67.2 were generated in our laboratory, as described previously [49]. Antibodies related to FLAG (A2220, Anti-Flag M2 Affinity Agarose Gel) were produced by Sigma-Aldrich, St. Louis, MO, USA.

## Data Availability

All data generated or analyzed during this study are included in this published article and its Supplementary Materials files.

## Supplementary Materials

The supporting information can be downloaded at: https://www.mdpi.com/article/10.3390/ijms25094617/s1.
