# Supplementary material for: Development of a New Model System to Study Long-Distance Interactions Supported by Architectural Proteins
Source: Int J Mol Sci. 2024 Apr 23;25(9):4617. doi: 10.3390/ijms25094617 (PMC11083095; doi:10.3390/ijms25094617)
Supplement: Supplementary file 1 [file ijms-25-04617-s001.zip › Supplementary information for Long Dist.pdf]

Supplementary information for

Development of a new model system to study long-distance interactions supported by architectural proteins

Larisa Melnikova [1,*], Varvara Molodina [1], Pavel Georgiev [2], Anton Golovnin [1,*]

[1] Department of Drosophila Molecular Genetics, Institute of Gene Biology, Russian Academy of Sciences, 34/5 Vavilov Street, 119334, Moscow, Russia; lsm73@mail.ru (L.M.); molodina_varvara@mail.ru (V.M.); agolovnin@mail.ru (A.G.)

[2] Department of the Control of Genetic Processes, Institute of Gene Biology, Russian Academy of Sciences, 34/5 Vavilov Street, Moscow 119334, Russia; georgiev_p@mail.ru (P.G.)

*****Correspondence: agolovnin@mail.ru (A.G.); lsm73@mail.ru (L.M.)

Table S2. Primer sequences used in ChIP–qPCR analysis

Primer pair	Sequences
62D-Fw<br>62D-Rev	5’ TTTGGGCTTGGTGAGAACAG 3’<br>5’ TGATACCAGGCGAACAGAAATC 3’
S^{x4}-Fw<br>S^{x4}-Rev	5’ GAGGGCTGCAGGAATTCGATG 3’<br>5’ CCTCTAGCGATAAGCTTGATCTAG 3’
Ras-Fw<br>Ras-Rev	5’ GAGGGATTCCTGCTCGTCTTCG 3’<br>5’ GTCGCACTTGTTACCCACCATC 3’

Supplementary Figures

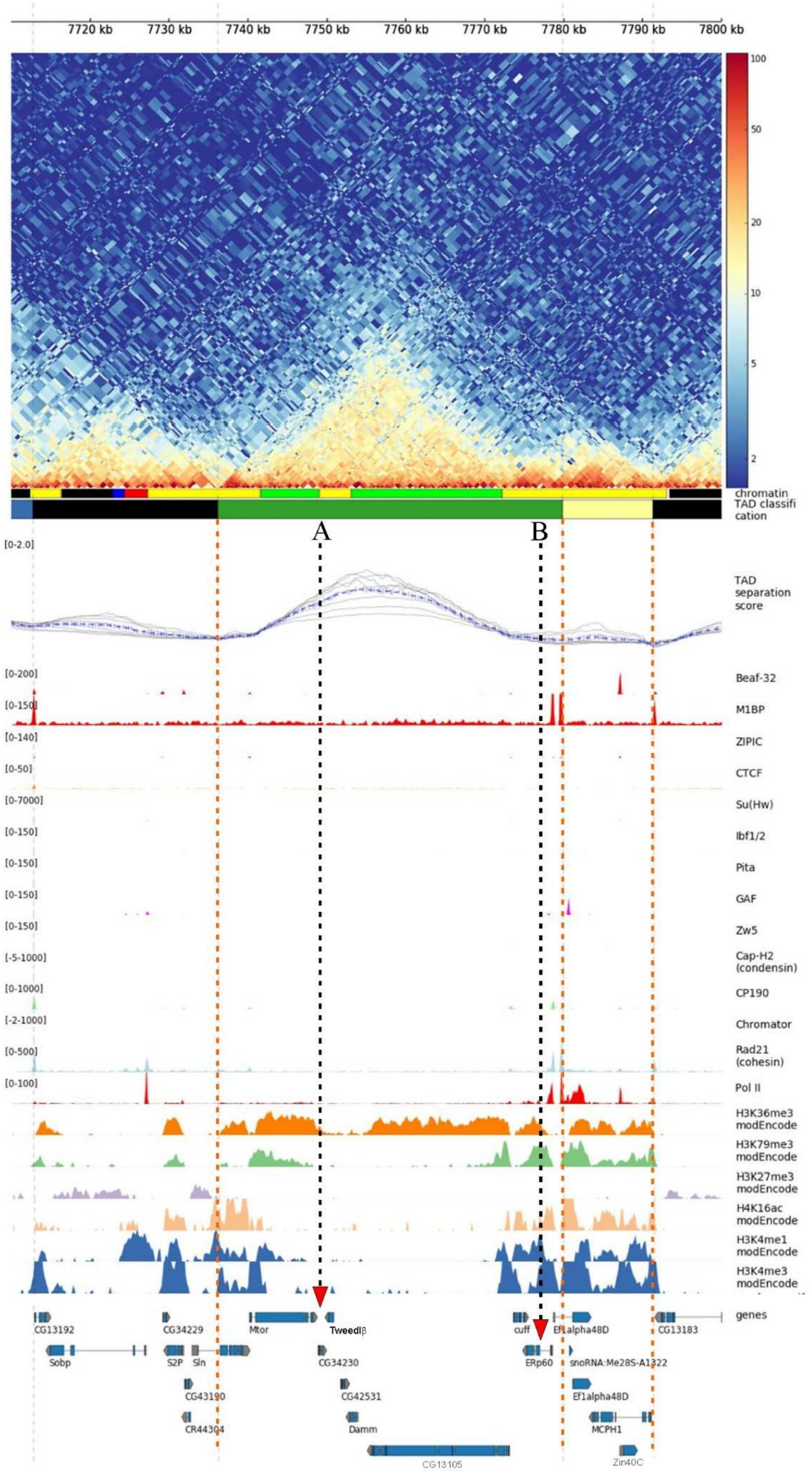

Figure S1. Genome region selected to create the model system. Chorogenome Navigator (http://chorogenome.ie-freiburg.mpg.de/) snapshot of selected 80 kb regions (2R:7720–7800 kb, scale indicated). The Hi-C map (interaction heat map indicated on the right), chromatin classification, genome-wide mapping information for various insulator and chromatin proteins (Beaf-32, M1BP, ZIPIC, CTCF, Su(Hw), Ibf1/2, Pita, GAF, Zw5, Cap-H2 (condensin), CP190, Chromator, Rad21 (cohesin), Pol II) and histone modifications (H3K36me3, H3K79me3, H3K27me3, H4K16ac, H3K4me1, H3K4me3), and gene models are shown. See Chorogenome Navigator (http://chorogenome.ie-freiburg.mpg.de/) for details. Orange punctuated vertical lines indicated TADs borders. Insertions sites in locus A (*CG34230*/*Tweedlβ* (2R:11862296)) and locus B (*ERp60* (2R:11890256)) are indicated by red triangles.

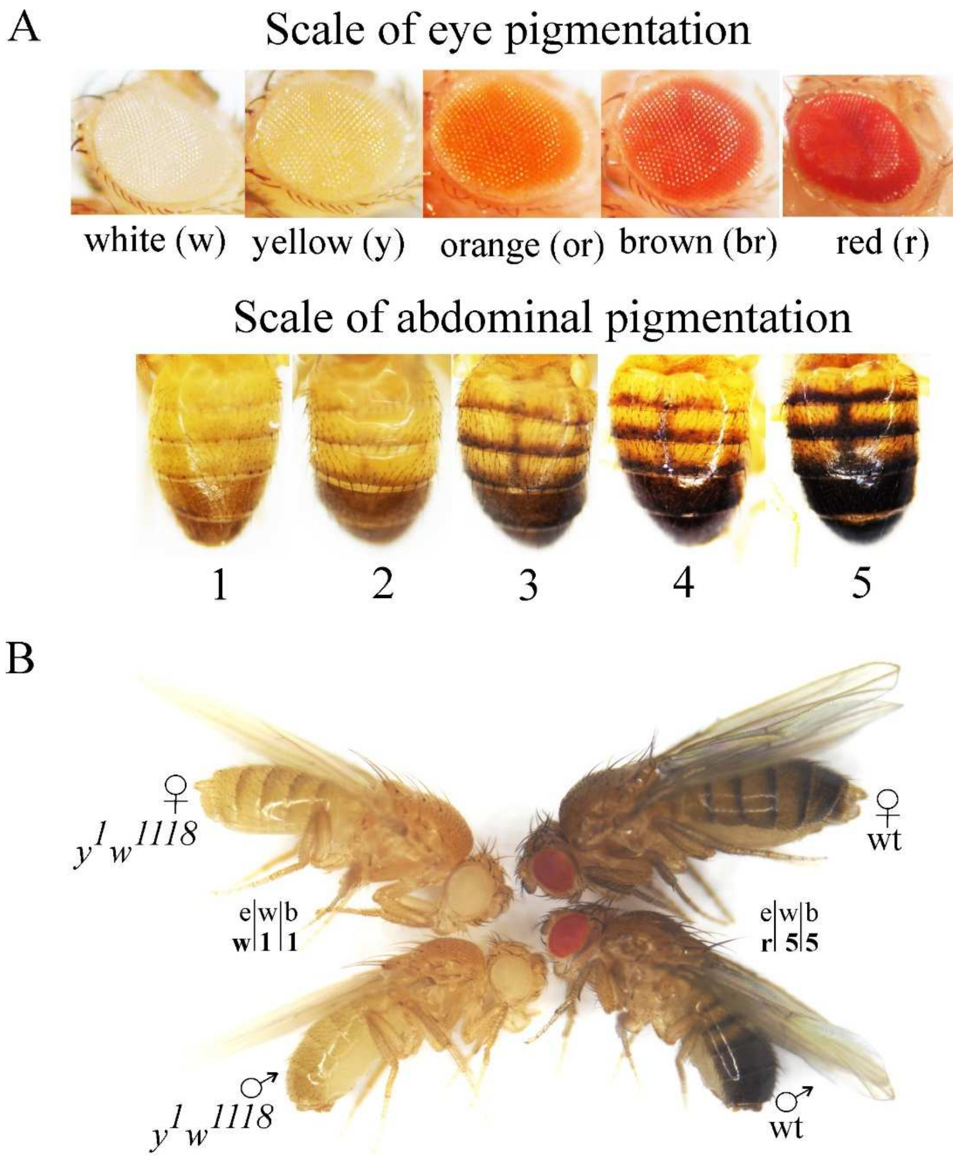

Figure S2. Analysis of *yellow* and *white* phenotypes. **(A)** Examples of eyes and abdominals pigmentation. Photos represent the eyes and abdominals pigmentation in the 3 d old males. Wild type *white* gene expression determined the bright red eye color (r); in the absence of *white* expression, the eyes are white (w). Intermediate levels of pigmentation are yellow (y), orange (or), and brown (br). Numbers indicate the scores of *yellow* gene expressions in the abdominal segments, which ranged from 1 (pigmentation as in y^1 allele) to 5 (pigmentation as in wild type flies). **(B)** Fly phenotypes from the y^1w^{1118} and wild type lines. Numbers next to the photo indicate the scores of *white* and *yellow* genes expression in the eyes (e), wings blades (w) and body cuticle (b).

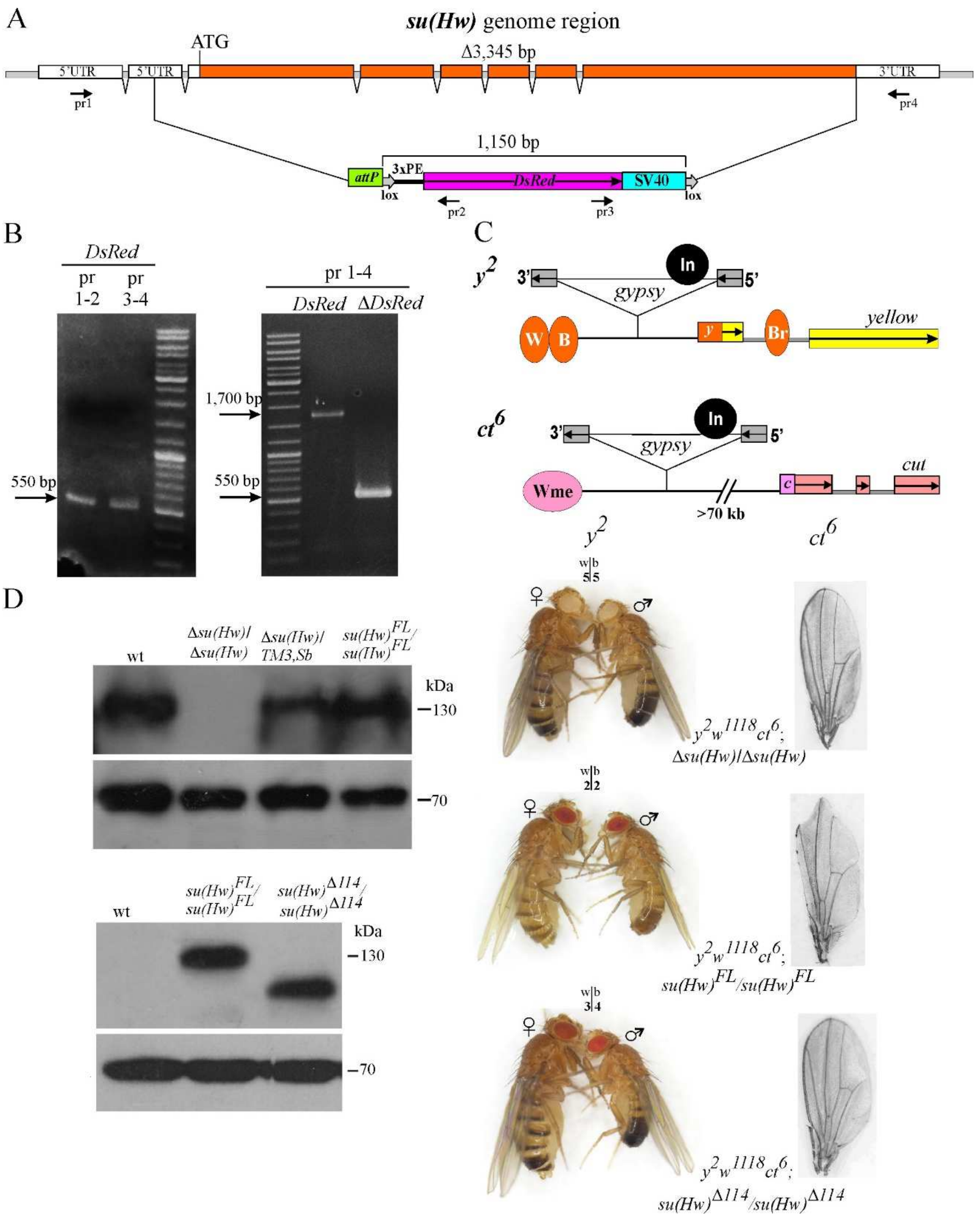

Figure S3. Functional analysis of the Su(Hw) protein derivatives. **(A)** A null mutation generated in the gene encoding the Su(Hw) protein using the CRISPR/Cas9 genome editing approach. Scheme (not to scale) of the *su(Hw)* gene with exons (orange rectangles) and introns between them. A 3345 bp sequence, including all coding regions, was deleted (Δ3345 bp) and substituted by the *loxP*-flanked *DsRed* reporter under the control of the 3xPE promoter (all designated). SV40 is a transcription terminator from the SV40 virus. Arrows show the positions and directions of primers (pr) 1 – 5′ ACCGATCCTCCACGTCCTTC 3′, 2 – 5′

CTCGAACTCGTGGCCGTTCA 3′, 3 – 5′ GTGGACTCCAAGCTGGACATCA 3′ and 4 – 5′ GTCAATGCGCAAGATGTCTGAAT 3′ used in the PCR. **(B)** PCR analysis of flies homozygous for Δ*su(Hw)*. PCR was performed before (*DsRed*) and after (Δ*DsRed*) deleting the *3xPE–DsRed–SV40* sequence by Cre-mediated recombination. PCR products on the left panel confirm the presence of *DsRed* cassette insertion. PCR products on the right panel confirm the presence of the *su(Hw)* deletion. **(C)** Scheme (not to scale) of the y^2 and ct^6 alleles. Exons in the *yellow* gene are shown as yellow rectangles. The *yellow* wing (W), body (B), and bristle (Br) enhancers are shown as ovals. Exons in the *cut* gene are shown as pink rectangles, and the *cut* wing margin enhancer (Wme) is shown as a magenta oval. The direction of gene transcription is indicated by black horizontal arrows. The *gypsy* insertion is shown as a triangle, in which the black circle (In) is the *gypsy* insulator, and the grey boxes are long terminal repeats, with arrows indicating their directions. The images show the loss of enhancer blocking in the homozygous Δ*su(Hw)* mutant background, the rescue of insulation by full-length Su(Hw) (Su(Hw)FL) expression, and the effect of Su(Hw)Δ114 expression on y^2 and ct^6 alleles (Su(Hw)Δ114). Enhancer–promoter interactions were analyzed for *yellow* and *cut* expression. Numbers above the photos indicate the scores of *yellow* genes expression in the wings blades (w) and body cuticle (b). Scales of abdominal pigmentation are presented in Figure S2A. **(D)** Western blot analysis of flies: $y^2w^{1118}ct^6$ (wt), homozygous for Δ*su(Hw)*, heterozygous for Δ*su(Hw)* (Δ*su(Hw)*/*TM3,Sb*), and expressing either Su(Hw)FL or truncated Su(Hw)Δ114 proteins tagged with the FLAG epitope. The protein extract was prepared from adult flies as described previously [1], resolved by electrophoresis in 7.5% SDS-PAAG, electroblotted onto a PVDF membrane, and probed with antibodies against Su(Hw) (α-Su(Hw), top panel) or against FLAG (α-FLAG, bottom panel). Anti-lamin staining (α-lamin) was used as the loading control. Molecular weights in kDa are marked on the right. The absence of the bands in the mutant Δ*su(Hw)*/Δ*su(Hw)* line, compared to the wt line, confirmed the presence of the deletion.

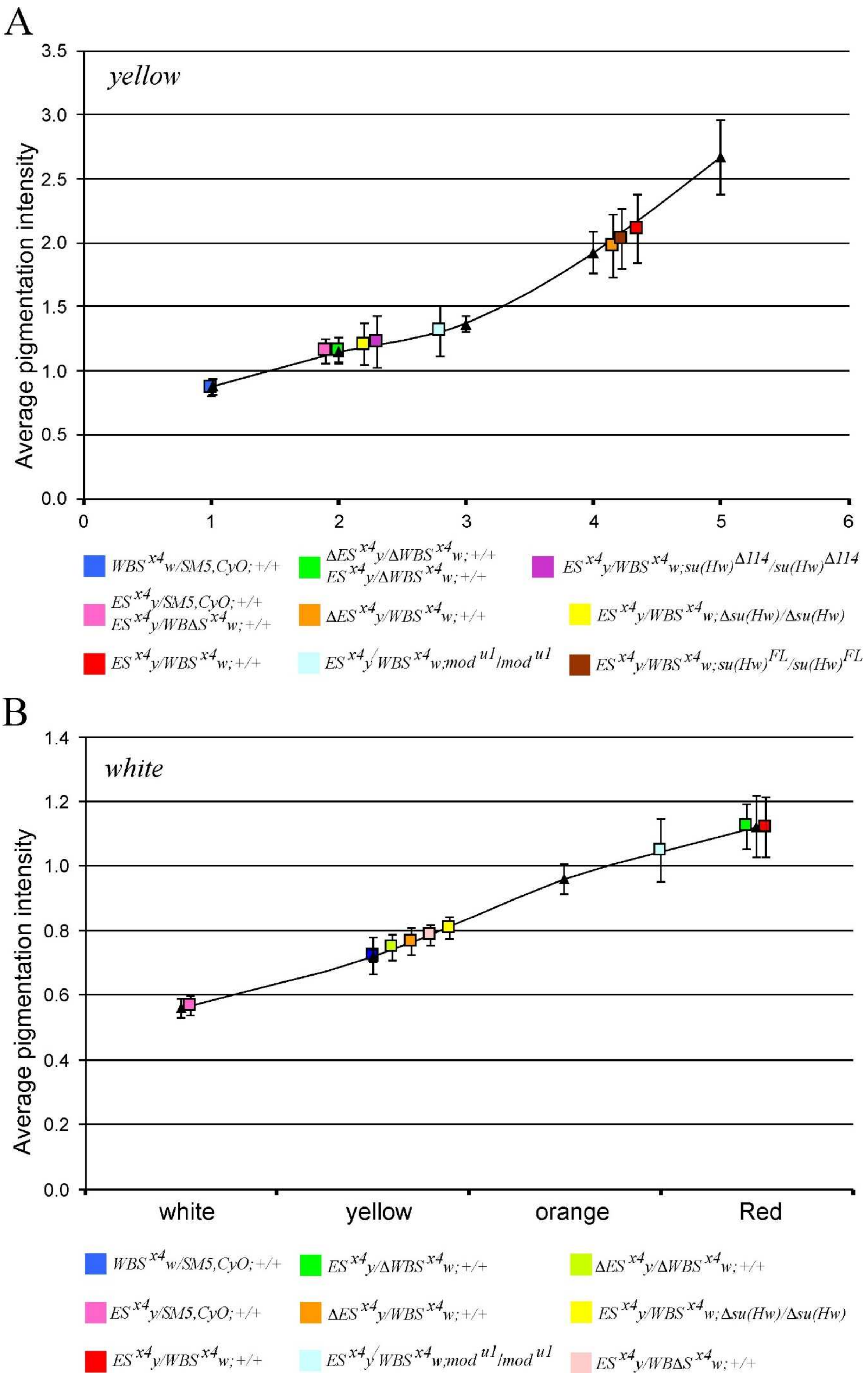
A
yellow
Average pigmentation intensity
0.0
0.5
1.0
1.5
2.0
2.5
3.0
3.5
0
1
2
3
4
5
6
WBS x4 w/SM5,CyO; +/+
ΔES x4 y/ΔWBS x4 w; +/+
ES x4 y/ΔWBS x4 w; +/+
ES x4 y/WBS x4 w;su(Hw) Δ114 /su(Hw) Δ114
ES x4 y/SM5,CyO; +/+
ES x4 y/WBΔS x4 w; +/+
ΔES x4 y/WBS x4 w; +/+
ES x4 y/WBS x4 w; Δsu(Hw)/Δsu(Hw)
ES x4 y/WBS x4 w; +/+
ES x4 y/ WBS x4 w;mod u1 /mod u1
ES x4 y/WBS x4 w; su(Hw) FL /su(Hw) FL
B
white
Average pigmentation intensity
0.0
0.2
0.4
0.6
0.8
1.0
1.2
1.4
white
yellow
orange
Red
WBS x4 w/SM5,CyO; +/+
ES x4 y/ΔWBS x4 w; +/+
ΔES x4 y/ΔWBS x4 w; +/+
ES x4 y/SM5,CyO; +/+
ΔES x4 y/WBS x4 w; +/+
ES x4 y/WBS x4 w; Δsu(Hw)/Δsu(Hw)
ES x4 y/WBS x4 w; +/+
ES x4 y/ WBS x4 w;mod u1 /mod u1
ES x4 y/WBΔS x4 w; +/+

Figure S4. Summarized data from the quantitative phenotypic analysis of reporter gene expression. The expression level is indicated by average pigmentation intensity (see Materials and Methods) for *yellow* **(A)** and *white* **(B)** genes. Numbers on the x-axis in the "*yellow*" panel indicates the scores of *yellow* expressions in the body cuticle, which range from 1 (no expression) through 2 (pigmentation as in the y^2 allele) to 5 (pigmentation as in wild-type males) (Figure S2A). Numbers on the x-axis in the "*white*" panel indicate the level of *white* gene expression in males' eye, which ranges from white (no expression) to red (pigmentation as in wild-type flies) (Figure S2A). Average pigmentation intensity (y-axis) is shown as an inverse number of RGB color values (0 to 255) multiplied by 100. The black curve with triangles in both panels represents the quantification of reference phenotypes. Colored boxes reflect the expression level of reporter genes in the corresponding construct combinations (indicated below panels). In the "*yellow*" panel **(A)**, genotypes with the same phenotypic manifistation are indicated by the same color. Error bars indicate the standard deviation of pigmentation intensity. Statistical material is present in supplementary data (Table S1) where pointed out that black curve fits were constructed by using excel Microsoft tools. Mutant background abbreviations: "+" – +/+, "*Δsu(Hw)*" – *Δsu(Hw)/Δsu(Hw)*, "$su(Hw)^{FL}$" – $su(Hw)^{FL}/su(Hw)^{FL}$, and "$su(Hw)^{\Delta 114}$" – $su(Hw)^{\Delta 114}/su(Hw)^{\Delta 114}$.

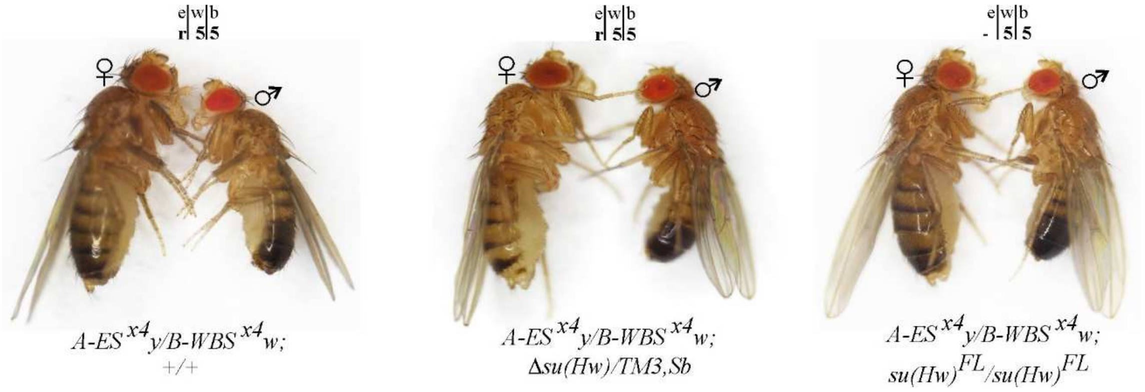

Figure S5. Fly phenotypes from the *A-ES*$^{\times4}$*y/B-WBS*$^{\times4}$*w* line with different mutant backgrounds. Numbers above the photo indicate the scores of *white* and *yellow* genes expression in the eyes (e), wings blades (w) and body cuticle (b). Sign "-" indicates that eye pigmentation was not scored. Scales of eye and abdominal pigmentation are presented in Figure S2A.

References

1. Golovnin, A.; Melnikova, L.; Babosha, V.; Pokholkova, G.V.; Slovohotov, I.; Umnova, A.; Maksimenko, O.; Zhimulev, I.F.; Georgiev, P. The N-Terminal Part of Drosophila CP190 Is a Platform for Interaction with Multiple Architectural Proteins. *Int J Mol Sci* **2023**, *24*, 15917, doi:10.3390/ijms242115917.
